# Real-time gap-free dynamic waveform spectral analysis with nanosecond resolutions through analog signal processing

**DOI:** 10.1038/s41467-020-17119-2

**Published:** 2020-07-03

**Authors:** Saikrishna Reddy Konatham, Reza Maram, Luis Romero Cortés, Jun Ho Chang, Leslie Rusch, Sophie LaRochelle, Hugues Guillet de Chatellus, José Azaña

**Affiliations:** 1 Institut National de la Recherche Scientifique—Énergie, Matériaux et Télécommunications (INRS-EMT), 800 de la Gauchetière Ouest, Suite 6900, H5A 1K6 Montréal Québec, Canada; 2 Centre for Optics, Photonics and Lasers (COPL), Department of Electrical and Computer Engineering, Université Laval, Quebec, G1V 0A6 Canada; 3 Université Grenoble Alpes/CNRS, LIPHY F-38000 Grenoble, France; 4 Present Address: Fonex Data Systems Inc., Montréal, Québec H4S 1P6 Canada

**Keywords:** Electrical and electronic engineering, Fibre optics and optical communications, Microwave photonics

## Abstract

Real-time tracking of a waveform frequency content is essential for detection and analysis of fast rare events in communications, radar, radio astronomy, spectroscopy, sensing etc. This requires a method that can provide real-time spectrum analysis (RT-SA) of high-speed waveforms in a continuous and gap-free fashion. Digital signal processing is inefficient to perform RT-SA over instantaneous frequency bandwidths above the sub-GHz range and/or to track spectral changes faster than a few microseconds. Analog dispersion-induced frequency-to-time mapping enables RT-SA of short isolated pulse-like signals but cannot be extended to continuous waveforms. Here, we propose a universal analog processing approach for time-mapping a gap-free spectrogram −the prime method for dynamic frequency analysis− of an incoming arbitrary waveform, based on a simple sampling and dispersive delay scheme. In experiments, the spectrograms of GHz-bandwidth microwave signals are captured at a speed of ~5×10^9^ Fourier transforms per second, allowing to intercept nanosecond-duration frequency transients in real time. This method opens new opportunities for dynamic frequency analysis and processing of high-speed waveforms.

## Abstract (web summary)

Real-time dynamic Fourier analysis of high-speed signals is difficult using either digital or analog schemes. Here, the authors present a universal analog approach that can track the changing frequency spectrum of waveforms in a real-time fashion at the nanosecond level, continuously and with no gaps.

## Introduction

Fourier spectral analysis of high-speed (broadband) time-varying waveforms, from the microwave to the optical domain, is a fundamental tool for a myriad of scientific and technological fields, e.g., for the characterization and manipulation of high-speed signals in communications, radar, and lidar systems^1–13^, observation of complex wave dynamics^14,15^, testing and design of ultrafast instrumentation, as well as for many spectroscopy, imaging, and sensing operations^16^, and radio astronomy research^17–20^. Many of these applications require that the desired frequency-domain information of the signal under test (SUT) is provided in a real-time dynamic fashion, e.g., so that to be able to track and/or manipulate the SUT frequency content as this evolves along the time domain, so-called real-time spectrum analysis (RT-SA)^21,22^. A key capability of an RT-SA system is that it should offer the possibility of capturing and identifying the frequency spectrum of fast rare or transient events purely in real time. For this purpose, one requires implementation of the RT-SA in a continuous and gap-free fashion, so that there are no dead times in acquisition or in processing the SUT. Otherwise, critical information may be lost in the waveform analysis process. Specific applications that require this stringent set of specifications include detection of radio frequency (RF) interferences or error signals in crowded wireless communications, such as for radar warning and electronic intelligence systems^2,4,5^, electronic countermeasure^3^, and wideband spectrum sensing^6^, as well as for detection of relevant frequency transients in modern biomedical instrumentation^11^ and radio astronomy^17–20^. These applications necessitate the capability to track or identify spectral events or transients with durations as short as a few nanoseconds or even faster^2–5,14–20^.

Fourier analysis is most often carried out by detection of the waveform of interest followed by analog-to-digital conversion (ADC) and subsequent electronic digital signal processing (DSP), involving computationally intensive fast Fourier transform (FFT) algorithms^20–26^. RT-SA based on this approach is significantly constrained by the performance (e.g., speed) limitations of present DSP engines^23,24^. In particular, presently available DSP-based RT-SA methods are limited to instantaneous operation bandwidths below the GHz regime (typically < 500 MHz), and they cannot intercept transients that are faster than a few microseconds with a 100% probability^21,22^. Alternatively, several analog wave processing approaches have been developed and utilized over the years^7–11^. A well-established strategy involves spatial dispersion of the desired spectral information, such as in standard optical spectral analysis^1,7,8^. However, spectral measurements based on spatial decomposition offer very limited update rates (i.e., constrained by the refresh rate of the spatial detection system), typically below the kHz range ^1^, thus being unsuited for the analysis of rapidly changing signals. Dynamic Fourier analysis can be carried out through characterization of consecutive time-windowed sections of the waveform, but this is restricted to repetitive signals and it is intrinsically slow^27,28^. Continuous gap-free spectral analysis of microwave signals has been also reported using complex light-wave spatial dispersion strategies^9^, though with temporal resolutions that are inherently limited by the detection refresh rate to at least a few tens of microseconds.

**Fig. 1:**
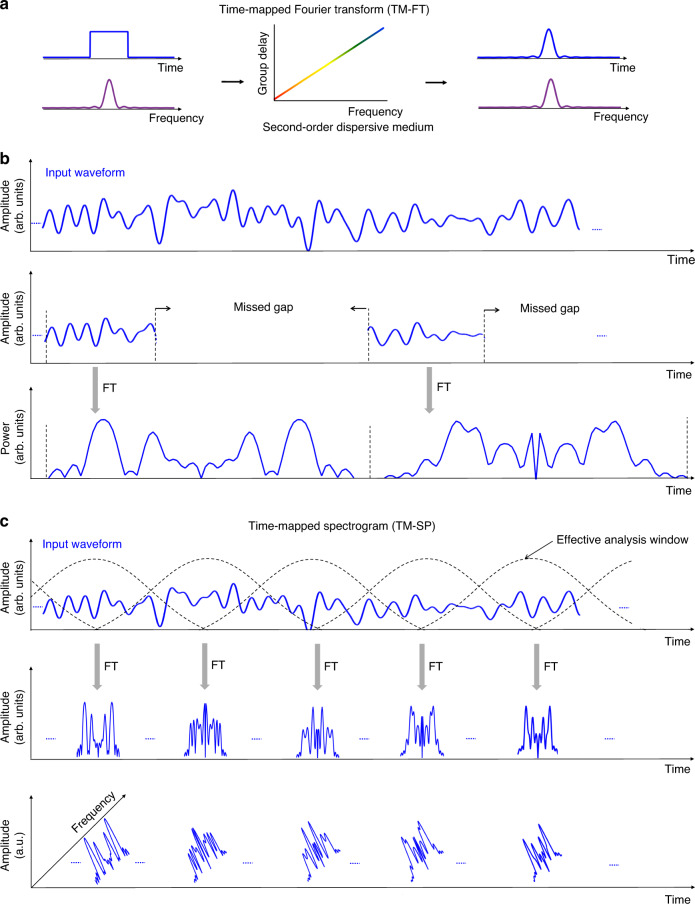
Illustration of the principles of time-mapped Fourier transform and the proposed time-mapped spectrogram analysis. **a** Analysis of a temporal pulsed waveform using the dispersion-based TM-FT. Here, the sinc-shaped power spectrum of the incoming square shaped temporal waveform, is mapped onto the output temporal domain after the TM-FT operation. **b** Analysis of a continuous temporal waveform using the dispersion-based TM-FT. The signal under analysis needs to be temporally truncated and subsequently stretched along the time axis, using linear chromatic dispersion. As a result, most of the signal information, in between consecutive truncated signal sections, is simply lost in this process. **c** A conceptual illustration of the proposed time-mapped spectrogram (TM-SP) analysis applied on the same incoming waveform. In this approach, the full short-time Fourier transform (ST-FT) distribution (in amplitude and phase), or full spectrogram, of the incoming temporal waveform is mapped along the temporal domain purely in real-time and in a continuous fashion. The TM-SP effectively performs Fourier analysis of consecutive time-windowed sections of the incoming waveform. This is achieved through a combination of short-pulse sampling and dispersive delay (see details in Fig. 2) without using an actual truncation or windowing of the incoming waveform. The mapped spectrogram involves the analysis of time-windowed consecutive sections of the signal under test that are heavily overlapped, which ensures that the continuous real-time spectral analysis is entirely gap-free.

The development of the time-stretch or time-mapped FT (TM-FT)^29–34^ has represented a fundamental milestone in the field of broadband spectral analysis. In its most basic form, the TM-FT uses linear chromatic (or group-velocity) dispersion to directly map the incoming wave spectrum along the time domain, so-called frequency-to-time mapping^31,32^, see illustration in Fig. 1a. Recall that in a dispersive medium, different frequency components of an incoming wave travel at different speeds^31^, so that each spectral component can be mapped to a different time delay at the output of the medium, obtaining its TM-FT. This approach allows one to capture and/or process broadband spectral information, including ultrafast rare/transient events, in real time through available time-domain instrumentation. The concept has enabled many important advancements in science and technology^14–16,33–41^. Nonetheless, TM-FT is inherently constrained to implement the static FT of a pulse-like waveform, in such a way that consecutive pulses (e.g., with changing spectra) must be temporally separated by a gap much longer than their individual pulse duration^31^. Such restrictive conditions are only satisfied by a limited sub-set of broadband signals, most typically, femtosecond/picosecond optical pulses with an inter-pulse spacing of at least a few nanoseconds^33,34^. In the most general case of continuous waveform analysis, the SUT needs to be truncated and subsequently stretched along the time axis, see illustration in Fig. 1b. As a result, most of the signal information in between consecutive truncated signal sections is simply lost in this process. Indeed, a conventional implementation of the method, enabling FT with a number of points of ~10, incurs in over 90% of signal loss (see the Section “Methods”). Alternative schemes have been demonstrated for TM-FT of broadband waveforms, e.g., by incorporating temporal modulation to the signal under analysis before dispersion^42–45^, or by exploiting the properties of frequency-shifted feedback loops^46^, but only static spectral analysis of time-truncated waveforms^42,43^ or strictly periodic signals^44–46^ has been reported through these methods. Thus, gap-free RT-SA of broadband arbitrary waveforms, with instantaneous frequency bandwidths exceeding the GHz range, remains very challenging.

Here, we propose and experimentally demonstrate a universal analog-processing concept for continuous and gap-free RT-SA of broadband waveforms. The proposed approach, herein referred to as the TM spectrogram (TM-SP), implements a direct, continuous mapping of a gap-free short-time FT (STFT) or SP—the prime tool for dynamic Fourier analysis^48–51^—of the incoming SUT along the time domain, see illustrated principle in Fig. 1c. This is achieved through a suitable combination of short-pulse sampling of the SUT followed by linear dispersive propagation, without actually implementing a moving time truncation of the incoming signal. We experimentally demonstrate TM-SP analysis of broadband (GHz-bandwidth) microwave signals using photonic sampling and optical dispersion. Through the demonstrated setup, the full SP of the incoming SUT is mapped along the time domain at a speed of ∼5 × 10^9^ FTs per second. Moreover, using this approach, we intercept and track arbitrary frequency transients with durations down to ~5 ns in real time. The performance of this proof-of-the-concept experimental demonstration is already well beyond the potential of current Fourier analysis methods and fulfills important requirements for RT-SA across a wide range of applications in communications, radar, radio astronomy, and others^1–26^.

## Results

### Concept and operation principle

**Fig. 2:**
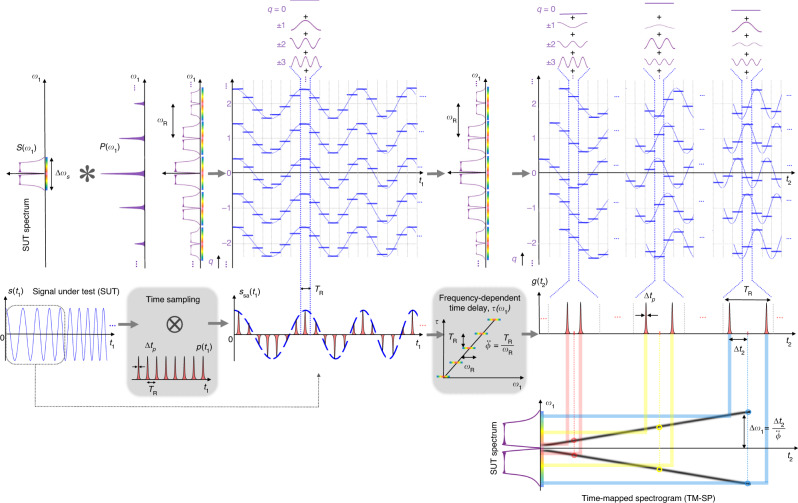
Basic principle of the proposed time-mapped spectrogram method. The scheme involves (i) a temporal sampling unit (first shaded box), where the signal under test (SUT), with a complex temporal envelope defined by *s*(*t*_1_), is modulated by a repetitive train of short pulses, with a sampling period defined by *T*_R_. Temporal sampling generates frequency-shifted copies of the input SUT, where each spectral copy is shifted by *ω*_R_ = 2*π*/*T*_R_. The temporal sampling is followed by (ii) a frequency-dependent (dispersive) time-delay unit (second shaded box), *τ*(*ω*_1_), where frequency shifted spectral copies (by *ω*_R_) are delayed with respect to each other by an amount *T*_R_. This second unit can be implemented through a dispersive element providing a linear group delay (as a function of radial frequency) with a slope defined by the parameter $\ddot \phi = \pm \!T_{\mathrm{R}}/\omega _{\mathrm{R}} = \pm \!T_{\mathrm{R}}^2/2\pi$ (dispersion), over the full frequency bandwidth of the sampling pulses. As illustrated in the top section of the figure, the interference between the simultaneously time delayed and frequency shifted copies of the SUT produces a temporal pattern *g*(*t*_2_) along each sampling-period slot (*T*_R_) that is proportional to the FT of the corresponding time-windowed section of the input signal, thus implementing a continuous time mapping of the signal′s short-time Fourier transform or spectrogram. In the example shown here, the SUT is a sinusoidal function with a frequency that is linearly increasing along time, so-called a linear frequency chirp signal. Thus, at the output of the proposed scheme, the FT of the corresponding time-windowed section of the SUT—namely two finite-duration pulses, corresponding to the double-side spectrum of a single-frequency sinusoidal function—is mapped along the time domain within each analysis period of duration *T*_R_. The obtained FT changes period by period—i.e., with an inter-pulse separation 2 × Δ*t*_2_ (proportional to twice the corresponding sinusoidal frequency) that increases linearly period by period—as the temporal analysis window is “virtually” shifted by this same delay (*T*_R_).

The TM-SP employs a very simple architecture, involving short-pulse temporal sampling and dispersive delay, according to the design conditions detailed below and as illustrated in Fig. 2. This scheme produces an output temporal waveform that follows the full complex STFT (in amplitude and phase) of the input signal, purely in real time and with a latency that is only limited by the propagation delay through the analog processing system. The combination of temporal sampling and dispersive delay performs a “virtual” temporal windowing of the input SUT and subsequent FT computation, without the need to implement an actual high-speed time truncation of the incoming signal. This is performed in such a way that consecutive analysis temporal windows overlap heavily along the signal of interest, thus ensuring that no signal information is lost (gap-free operation).

Let the incoming temporal waveform or SUT generally be expressed as $s\left( {t_1} \right){\mathrm{e}}^{\left( { i\omega _0t_1} \right)}$, where *t*_1_ is the time variable, *s*(*t*_1_) is the signal’s complex temporal envelope, *i* is the imaginary unit and *ω*_0_ is the signal’s central (or carrier) frequency. The SUT is assumed to be band-limited with a full frequency bandwidth denoted by Δ*ω*_*s*_. No additional constraints are imposed on the signal’s time duration (e.g., the SUT can be infinitely long). The FT of the signal’s complex envelope *s*(*t*_1_) is defined as *S*(*ω*_1_) = FT{*s*(*t*_1_)}, where *ω*_1_ holds for the radial frequency variable. The STFT of the SUT is a FT of consecutive time-windowed signal sections, defined as^48^

1 $${\mathrm{STFT}}_s\left( {\tau ,\omega _1} \right) 	= {\mathrm{FT}}\left\{ {h\left( {t_1 - \tau } \right)s\left( {t_1} \right)} \right\} \\ 	= \int _{ - \infty }^\infty h\left( {t_1 - \tau } \right)s\left( {t_1} \right)e^{\left( { - i\omega _1t_1} \right)}dt_1,$$

where *h*(*t*) is a time-limited function that serves as the temporal window for calculation of the STFT. The SP of the signal is then, SP_*s*_(*τ*, *ω*_1_) = |STFT_*s*_(*τ*, *ω*_1_)|^2^.

As illustrated in Fig. 2, the proposed TM-SP method involves a cascade of (i) a conventional temporal sampling unit to modulate the SUT’s envelope, *s*(*t*_1_), with a periodic train of short pulses, and (ii) a frequency-dependent time delay unit, which can be practically implemented, e.g., using chromatic dispersion^30–34^. The sampling pulses in unit (i) are defined by a time-limited function *p*(*t*_1_) (individual pulse shape), repeating with a period *T*_R_ = 2*π*/*ω*_R_, where *ω*_R_ is the sampling frequency. The sampling process must satisfy the well-known Nyquist criterion, i.e., the sampling frequency must be higher than the SUT’s full bandwidth, *ω*_R_ > Δ*ω*_*s*_^52^. This process produces copies of the SUT’s spectrum along the frequency domain, spaced by the sampling frequency. The frequency-dependent time delay unit delays each of these spectral copies by an amount Δ*τ* with respect to each other, as represented in Fig. 2. A key design condition is that this relative delay must be equal to the sampling period, i.e., Δ*τ* = *T*_R_. Thus, the slope of the dispersive delay process must satisfy the following rule:

2 $$\left| {\ddot \phi } \right| = \frac{{\Delta \tau }}{{\omega _{\mathrm{R}}}} = \frac{{T_{\mathrm{R}}^2}}{{2\pi }}.$$

The system produces an interference of consecutive delayed and frequency-shifted copies of the original signal, resulting in a time-domain waveform that follows the STFT of the incoming SUT, see general concept illustrated in Fig. 1c and implementation details in Fig. 2. In particular, this self-induced wave interference leads to the computation of the FT of consecutive time-windowed sections of the SUT, in such a way that the resulting spectra are mapped along consecutive time slots, each with a duration equal to the sampling period, *T*_R_. Briefly, as illustrated in Fig. 2, a set of harmonically related frequency components are coherently added along each sampling-period slot, *T*_R_ in such a way that these different frequency components are weighted by the amplitude and phase of a set of consecutive samples of the SUT, leading to the calculation of the FT of the corresponding time-windowed section of the input signal *s*(*t*_1_). The analyzed signal section is shifted by *T*_R_ from each sampling-period slot to the following one. As a result, this scheme produces an output temporal waveform that follows the signal STFT. Supplementary Fig. 1 shows a more detailed illustration of the mathematical relationships that lead to the analog computation and time mapping of the signal’s STFT, as described in what follows. We reiterate that the temporal windowing and FT computation processes are inherently provided by the combination of sampling and dispersive delay, under the given design conditions.

A detailed mathematical analysis of the proposed scheme is provided in the “Methods” section. For this analysis, we consider the output temporal waveform over consecutive time slots of duration *T*_R_, where the *n*th time slot (with *n* = 0, ±1, ±2…,) is defined by $nT_{\mathrm{R}} - \frac{{T_{\mathrm{R}}}}{2} \, \le \, t_2 \, < \, nT_{\mathrm{R}} + \frac{{T_{\mathrm{R}}}}{2}$, with *t*_2_ = *t*_1_ − Δ*t*_L_ being the time variable at the system output, simply delayed with respect to the input (*t*_1_) by the overall latency of the system, Δ*t*_L_. Consistently with the illustrations in Supplementary Fig. 1, our mathematical derivations prove that the complex temporal envelope of the output waveform at the *n*th time slot can be written as follows (using the STFT definition in Eq. (1)):

3 $$g_n\left( {t_2} \right) \propto {\mathrm{FT}}\left\{ {h\left( {t_1 - nT_{\mathrm{R}}} \right)s\left( {t_1} \right)} \right\} \equiv {\mathrm{STFT}}_s\left( {nT_{\mathrm{R}},\omega _1} \right),$$

with $\omega _1 = \frac{{t_2 - nT_{\mathrm{R}}}}{{\ddot \phi }}$ and $h\left( t \right) \equiv {\mathrm{FT}}\left\{ {p(t_1)} \right\}_{\omega _1 = \frac{{ - t}}{{\ddot \phi }}}$. Hence, Eq. (3) indicates that the proposed system inherently computes the STFT of the waveform under analysis, *s*(*t*_1_), namely the FT of a time-windowed version of the SUT, with the central location of the temporal analysis window, *h*(*t*), running as *nT*_R_, with *n* = 0, ±1, ±2,…. Furthermore, the obtained STFT is directly mapped along the time domain, consecutively within each of the analysis sampling-period slots. Specifically, the output temporal waveform along the sampling-period slot centered at *nT*_R_, [*g*_*n*_(*t*_2_)] is just a TM copy of the corresponding STFT of the input signal, i.e., the STFT computed with a temporal window centered around *t*_1_ = *nT*_R_. As shown by Eq. (3), the frequency-to-time mapping factor (Δ*t*_2_ ← Δ*ω*_1_) is defined by the dispersion factor $\ddot \phi$, i.e., $\frac{{\Delta t_2}}{{\Delta \omega _1}} = \ddot \phi$, so that the entire frequency spectrum of the truncated signal is TM within the corresponding analysis period (duration *T*_R_).

The time resolution (*δt*_r_) of the obtained STFT distribution^47,48^ is dictated by the duration of the temporal analysis window function *h*(*t*). As defined by Eq. (3), the analysis window duration (Δ*t*_*h*_) is given by the frequency bandwidth of the sampling pulses (Δ*ω*_*P*_) scaled along the time domain by the factor $\ddot \phi$, so that $\delta t_{\mathrm{r}} \approx \Delta t_h \approx \left| {\ddot \phi } \right| \times \Delta \omega _p$. The uncertainty principle of the FT imposes that the frequency resolution of the obtained STFT is directly given by the inverse of the time resolution^47,48^, $\delta \omega _{\mathrm{r}} \approx \Delta t_p/\left| {\ddot \phi } \right|$. These estimates indicate that the resolution of the TM-SP (along the time axis) is directly determined by the temporal width of the sampling pulses, Δ*t*_*P*_. Considering that the full analysis frequency bandwidth is mapped along a single temporal sampling period, *T*_R_, each windowed Fourier spectrum is resolved in time with a total number of features given by the factor *M*≈*T*_R_/Δ*t*_*P*_. Moreover, it can be easily inferred that the temporal resolution of the computed STFT (duration of the analysis temporal window, *h*(*t*)) is about *M* times longer than the sampling period, i.e., *δt*_r_ ≈ Δ*t*_*h*_*≈* *M* × *T*_R_. Considering that the time shift between consecutive analysis windows in the SP calculation is given by the sampling period *T*_R_, see Eq. (3), this implies that consecutive temporal analysis windows are heavily overlapped, which ensures that the conducted RT-SA is gap-free, with no dead times in the acquisition or FT computation. In fact, the TM windowed spectrum keeps approximately the same (i.e., repeating) over the time resolution of the implemented SP, corresponding to about a number *M* of consecutive representation periods (each with a duration *T*_R_). This translates into an effective oversampling of the STFT information, which could be exploited in order to relax the specifications of the detection stage used for capturing the TM-SP distribution (further discussions below).

### Experimental demonstration

**Fig. 3:**
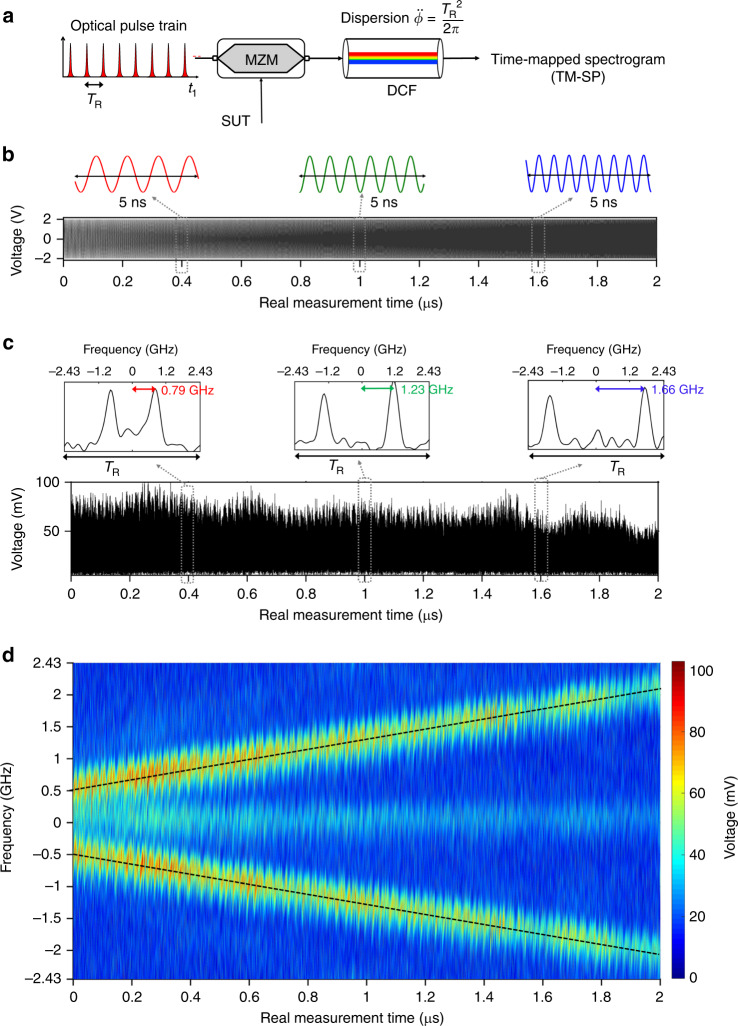
Experimental results on time-mapped spectrogram analysis of a linearly chirped RF waveform using photonic sampling and dispersion. **a** A schematic of the photonics-based TM-SP experimental setup for real-time spectral analysis of broadband microwave signals. The microwave signal under test (SUT) is sampled with a picosecond optical pulse train generated from a mode-locked fiber laser, in a Mach–Zehnder modulator (MZM). The optical samples are propagated through dispersion compensating fiber (DCF) with a total dispersion $\ddot \phi$, and the output TM-SP is captured in a real-time oscilloscope after photo-detection. **b** The temporal trace of the microwave signal under test (SUT), with a linearly increasing frequency, from 500 MHz to 2 GHz, along a duration of 2 µs. **c** The photo-detected output temporal waveform (voltage signal) that is directly captured in a real-time oscilloscope, over the same 2-µs duration. The inset plots show a zoom of the output waveform around three different time slots, each extending over one analysis period (*T*_R_ = 205.6 ps). The temporal trace along each consecutive analysis period, *T*_R_, is a time-mapped copy of the FT of the input SUT, effectively windowed around the corresponding time of analysis. The top horizontal axis follows the frequency scale that is obtained using the mapping law $\Delta t_2 \leftarrow \Delta \omega _1\ddot \phi$. The instantaneous spectrum of the input signal at any given instant of time consists of two individual pulses, corresponding to the frequency of the signal, ±*ω*_RF_(*t*_1_) linearly increasing with time. The observed signal background (including at the location corresponding to DC) is attributed to unwanted variations in the bias condition of the electro-optic modulation process with respect to the optimal design. **d** A 2D representation of the signal joint time–frequency energy distribution (spectrogram) that is directly recovered from the output temporal trace.

In order to experimentally verify the proposed TM-SP concept, we designed a simple photonics platform for gap-free RT-SA of GHz-bandwidth microwave signals, using widely available off-the-shelf photonics components. Customized high-speed microwave waveforms were generated using an electronic arbitrary waveform generator (AWG). The experimental setup follows the general schematic shown in Fig. 3a. A train of picosecond optical pulses, repeating at a rate of 4.86 GHz (*T*_R_ = 205.76 ps), modulates the microwave SUT in an electro-optic Mach–Zehnder modulator (MZM), implementing the temporal sampling unit. The linear dispersive delay is implemented by a length of dispersion compensating fiber (DCF), with a total dispersion value of ∼6825 ps^2^ rad^−1^. The output waveform is captured by an electronic real-time oscilloscope after optical-to-electrical conversion in a 50-GHz photo-detector. We reiterate that the proposed concept does not impose any fundamental limitation on the signal duration: the signal Fourier content is inherently time mapped in a continuous fashion as it propagates through the TM-SP system. The duration of each of the studied waveforms in our experimental examples was limited by the available memory of the instruments used for generation (AWG) and measurement (real-time scope) of waveforms. In addition, in this way, the entire output signal could be captured with the real-time scope for subsequent off-line analysis and representation of the detected data (e.g., for the 2D time–frequency graphs).

In the first example (results shown in Fig. 3), we present the analysis of a 2-μs long microwave signal with an instantaneous frequency *ω*_RF_(*t*_1_), linearly increasing from 500 MHz to 2 GHz, Fig. 3b. The plot in Fig. 3c shows the voltage of the output electrical temporal waveform that is directly captured on the real-time scope. The measured output temporal waveform along each slot of duration *T*_R_ (sampling period) is mapped into the equivalent frequency axis using the defined mapping law, $\Delta t_2 \leftarrow \Delta \omega _1\ddot \phi$. The instantaneous spectrum of the input SUT at any given instant of time consists of two individual pulses, corresponding to the instantaneous frequency of the signal, ±*ω*_RF_(*t*_1_) linearly increasing with time. Figure 3d shows the 2D representation of the signal joint time–frequency energy distribution (SP) that is directly recovered from the output temporal trace. As expected, the experimentally recovered SP follows the theoretical linear frequency chirp of the input SUT (dashed gray lines). The deviations of the 2D distribution along the vertical frequency axis, with respect to the theoretical linear frequency chirp, that are observed in Fig. 3d are attributed to the finite timing jitter in the picosecond optical pulses used for temporal sampling. It is important to highlight that no rescaling was applied to represent the time axis in the 2D time–frequency plot in Fig. 3d, which indicates that the output waveform delivers the SP of the input signal purely in real time. Specifically, the changing signal FT is calculated every *T*_R_ ≈ 205.76 ps, i.e., at a speed of 4.86 × 10^9^ FTs/s, significantly outperforming present instrumentation. For a comparative reference, a state-of-the-art real-time RF spectrum analyzer based on DSP, the Keysight N9040B-RT2, provides a maximum of 292,969 discrete FTs per second ^22^. The results in Fig. 3 confirm the capability of the demonstrated setup to provide a gap-free RT-SA of waveforms with an instantaneous frequency bandwidth approaching 5 GHz, beyond the capabilities of current electronic DSP-based platforms.

The frequency resolution estimate provided above is valid assuming that the detection bandwidth is high enough to resolve the sampling pulses; otherwise, one should rather consider the temporal width of the detected pulses for this estimate (Δ*t*_d_ ← Δ*t*_*p*_). In our particular experimental realization, the full-width at half-maximum (FWHM) of the sampling pulses was Δ*t*_*p*_ ∼ 7 ps, whereas the FWHM after photo-detection (i.e. in intensity) was measured to be about twice this value, Δ*t*_d_ ∼ 14.4 ps, see Supplementary Fig. 2. This corresponds with a theoretical frequency resolution $\delta \omega _{\mathrm{r}} \approx \Delta t_{\mathrm{d}}/\left| {\ddot \phi } \right|$ ~ 2*π* × 340.3 MHz. Note that the limited detection bandwidth does not modify the time resolution of the calculated SP, whose amplitude FWHM width is given by $\delta t_{\mathrm{r}} \approx \left| {\ddot \phi } \right| \times \Delta \omega _p\sim 5.9\,{\mathrm{ns}}$. Thus, a longer time resolution can be obtained by use of a sampling pulse with a broader frequency bandwidth, practically limited only by the operation bandwidth of the dispersive line used in the system. As mentioned, the frequency resolution will however remain limited by the detection bandwidth. The highest frequency resolution offered by the system (inverse of the SP time resolution) can be exploited only if a detection stage is available with a sufficiently large bandwidth to capture the sampling pulse time width (with no distortion). Additional experiments are reported in Supplementary Fig. 3 that validate further the derived time–frequency resolution estimates.

**Fig. 4:**
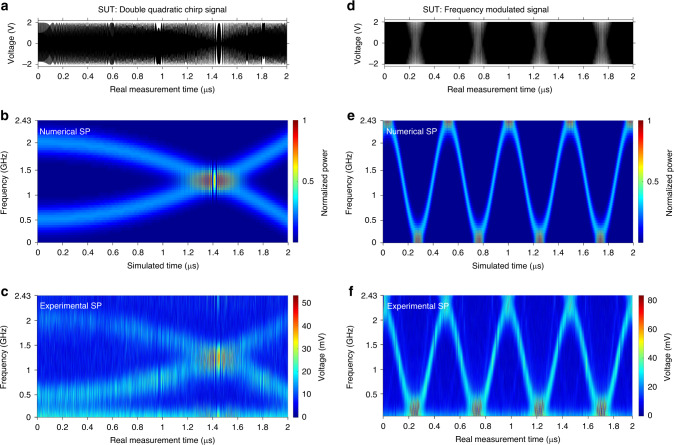
Experimental results on time-mapped spectrogram analysis of two complex high-speed microwave signals using photonic sampling and dispersion. **a**–**c** The results corresponding to the analysis of a 2-µs long microwave signal under test (SUT), which is composed by two superimposed frequency-chirped sinusoids, namely a sinusoid with an increasing quadratic frequency chirp, from 500 MHz to 2 GHz, and a sinusoid with the same but opposite frequency chirp. The signal exhibits two separate frequency bands at each instant of time. **d**–**f** The results corresponding to the analysis of a 2-MHz tone that is frequency modulated on a 1.215-GHz microwave carrier with a maximum frequency deviation of ±1.215 GHz. For each of the analyzed signals, the top plot, (**a**) and (**d**), respectively, shows the measured temporal waveform (voltage) of the input microwave SUT; the plot in the middle, (**b**) and (**e**), respectively, shows the numerically computed spectrogram (SP) distribution of the measured input signal, calculated using a 5.9-ns FWHM width Gaussian pulse as the analysis temporal window; and the bottom plot shows the 2D TM-SP distribution that is directly recovered from the measured temporal trace at the output of the experimental photonic sampling and dispersion scheme. To facilitate interpretation of the obtained results, in each spectrogram representation, only the positive axis of frequencies is represented.

Figure 4 reports the results of gap-free RT-SA of two additional, more complex GHz-bandwidth microwave signals using the described photonics platform: a signal composed by two superimposed sinusoidal waveforms with exact opposite quadratic frequency chirps (left plots), and a sinusoidally frequency-modulated carrier (right plots). For each of these testing cases, the top plot (a, d) shows the measured input temporal waveform; the plot at the center (b, e) shows the numerically computed SP of the digital version of the input SUT, using a temporal analysis window that matches the one implemented by the real-time photonics platform, i.e., a 5.9-ns (FWHM) Gaussian pulse; and the plot at the bottom (c, f) shows the 2D SP representation that is directly recovered from the measured output temporal trace. As expected, in all cases, there is an excellent agreement between the measured real-time SP and the numerically calculated one. The numerical SP required a heavy digital computation based on FFT algorithms and in particular, considering the specifications of the general-purpose computing platform used for these calculations, this was obtained at an estimated speed of ~6 FTs/s (see details in “Methods”). In sharp contrast, the reported photonic-based analysis platform provides the SP distribution purely in real time, at nearly ∼5 × 10^9^ FTs/s.

As mentioned above, there is a heavy overlapping between consecutive virtual temporal analysis windows, as inherently implemented by the TM-SP process. As a result, the TM windowed spectrum keeps nearly identical over a number of about *M* consecutive representation periods, each with a duration *T*_R_, where we recall that *M* ≈*T*_R_/Δ*t*_*p*_ (*M* ∼29 in our experimental design). Using this property, the obtained SP could be fully retrieved by sampling the output waveform at a significantly relaxed rate, just slightly below the original sampling rate, e.g., with a period equal to *T*_R_ + *T*_R_/*M*. This interesting property has been confirmed through numerical down-sampling of the output waveforms captured with the real-time scope in the reported experiments, see examples shown in Supplementary Fig. 4.

**Fig. 5:**
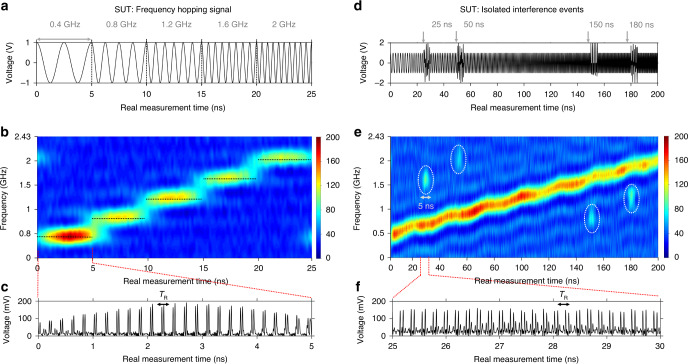
Experimental results on the capability of the time-mapped spectrogram analysis method to capture nanosecond-duration frequency transients along the incoming broadband microwave signal. **a** The temporal trace of a microwave signal under test (SUT) consisting of a fast frequency hopping sequence, designed to test the time-mapped spectrogram (TM-SP) method performance. The sequence is composed by consecutive 5-ns-long tones with linearly increasing hopping frequencies, i.e., 0.4, 0.8, 1.2, 1.6, and 2 GHz. **b** The 2D spectrogram distribution that is directly recovered from the measured temporal trace at the output of the TM-SP setup, involving photonic sampling and dispersion with the parameters given in the text. The hopping frequency speed that can be captured with this setup is only limited by the time resolution of the calculated TM-SP, whose FWHM width is 5.9 ns in these experiments. **c** A zoom of the output temporal waveform over the 5-ns temporal window corresponding to the presence of the 0.4-GHz tone, confirming that the resultant time-mapped spectral shape is recovered repeatedly at every *T*_R_ (=205.6 ps) along this analysis window. **d** The temporal trace of a microwave SUT composed by (i) a linearly increasing frequency chirp waveform, from 500 MHz to 2 GHz, and (ii) isolated interfering frequency transients with the same amplitude as that of the frequency chirp signal, each having a duration of 5 ns. The interfering frequency transients are centered at 1.6, 2, 0.8, and 1.2 GHz, occurring at the relative times of 25, 50, 150, and 180 ns, respectively. **e** The 2D spectrogram distribution that is directly recovered from the measured temporal trace at the output of the TM-SP setup. The TM-SP method is clearly able to capture and analyze the 5 ns duration isolated interfering frequency transients, consistently with the predicted temporal resolution of analysis in these experiments. **f** A zoom of the output temporal trace over the 5-ns temporal window corresponding to the 1.6-GHz interference event. To facilitate interpretation of the obtained results, in each 2D spectrogram representation, only the positive axis of frequencies is represented.

Finally, gap-free continuous RT-SA of broadband waveforms with nanosecond resolutions is also demonstrated through the experimental results shown in Fig. 5. The SUTs analyzed here include fast random or isolated events and frequency transients with durations down to 5 ns. Again, this is beyond the performance of present DSP-based RT-SA schemes, which are typically limited to provide 100%-probability interception of signal features with durations at least in the microsecond range^21,22^. Figure 5a shows the temporal trace of a fast microwave frequency hopping sequence that is purposely designed to test the TM-SP setup performance. In particular, the SUT consists of 5-ns long segments with a linearly increasing frequency, from 0.4 to 2 GHz. Figure 5b shows the 2D SP distribution that is directly recovered from the measured temporal trace at the output of the TM-SP setup, revealing the designed hopping sequence in the time–frequency plane. Figure 5c shows a zoom of the output temporal waveform over the 5-ns temporal window corresponding to the presence of the 0.4-GHz tone. This plot reveals that the TM spectrum of this event is produced repetitively over this 5-ns window at every sampling period (i.e., about 25 times), ensuring that the event is intercepted with 100% probability. As another example, Fig. 5d, e shows the results (input temporal trace and recovered SP, respectively) of the analysis of a microwave signal composed by a linearly increasing frequency chirp, along with random isolated interfering frequency transients, each having a duration of 5 ns. Figure 5f also shows a zoom of the output temporal trace over the 5-ns temporal window corresponding to the 1.6-GHz interference event, revealing again the oversampling features of the conducted RT-SA. These results clearly confirm that the photonic-based TM-SP platform allows one to intercept the frequency content of any random or isolated signal event in a real-time fashion as long as this occurs over a duration of the order of (or longer than) the time resolution of the conducted SP, ∼5.9 ns FWHM width in the reported experimental configuration.

## Discussion

In the proposed method (TM-SP), the full STFT, or SP, of the incoming waveform is mapped along the time domain in such a way that the output processed signal remains in the physical wave domain. As shown herein, this provides a simple and straightforward method for real-time analog characterization and tracking of the signal’s time-changing spectrum, in a continuous and gap-free fashion, overcoming key performance limitations of present RT-SA methods. The proof-of-concept experimental setup reported here already enables gap-free RT-SA of GHz-bandwidth waveforms, including the capability of intercepting random fast signals with durations down to the nanosecond regime. This performance fulfills critical requirements of RT-SA for a wide range of applications, in communications, radar, radio astronomy, biomedical instrumentation, and others. The proposed design for TM-SP is universal in that it is suited for analysis and processing of waveforms across any frequency region, provided the availability of technologies for temporal sampling and dispersive delay. Recent developments on mode-locked lasers with hundreds of GHz repetition rates^52^, matched by temporal modulation technologies with similar bandwidth performance^53^ and all-optical temporal sampling techniques for output waveform measurements^54^, offer the potential to enable sub-THz bandwidth RT-SA using the proposed method.

More generally, TM-SP analysis provides unprecedented capabilities to process the entire signal’s time–frequency distribution in an efficient and direct fashion, using well-established time-domain wave manipulation methods. In particular, we anticipate that important operations, such as time-varying (dynamic) frequency filtering at high speeds, re-shaping of a waveform to provide a prescribed time–frequency distribution (so-called time–frequency synthesis), or pattern identification in the joint time–frequency plane^47–50^, could be all implemented on-the-fly using readily available temporal processing techniques, e.g., temporal modulation on the TM-SP signal. These prospects are particularly interesting for broadband waveforms, i.e., with bandwidths in the GHz regime and above, from the microwave to the optical domain, for which real-time dynamic Fourier analysis and processing tools remain challenging, beyond the potential of present methods and technologies.

## Methods

### Derivation of the mathematical expression of the output TM-SP

As described, we assume that the temporal SUT can be generally expressed (analytical function) as $s\left( {t_1} \right){\mathrm{e}}^{\left( { i\omega _0t_1} \right)}$, where *s*(*t*_1_) is the signal’s complex temporal envelope, *t*_1_ stands for the time variable (system input), *ω*_0_ is the signal′s carrier frequency (*ω*_0_ = 0 for a base-band signal), and ${i} = \sqrt { - 1}$ is the imaginary unit. The SUT is assumed to be band-limited with a full frequency bandwidth denoted by Δ*ω*_*s*_. No constraints are imposed on the signal′s time duration, so that in general, this can be infinitely long $\left( { - \infty \, \le \, t_1 \, \le + \infty } \right)$. The FT of the signal′s complex envelope *s*(*t*_1_) is defined as

4 $$S\left( {\omega _1} \right) = {\mathrm{FT}}\left\{ {s(t_1)} \right\} = \int _{ - \infty }^\infty s\left( {t_1} \right){\mathrm{e}}^{\left( { - i\omega _1t_1} \right)}dt_1,$$

where *ω*_1_ holds for the radial frequency variable. By extension, the short-time FT (STFT) of the SUT is then mathematically defined by the following expression^48^:

5 $${\rm{STFT}}_s(\tau ,\omega _1) = {\mathrm{FT}}\left\{ {h(t_1 - \tau )s(t_1)} \right\} = \int _{ - \infty }^\infty h(t_1 - \tau )s\left( {t_1} \right){\mathrm{e}}^{\left( { - i\omega _1t_1} \right)}dt_1,$$

where *h*(*t*) is a time-limited function that serves as the “temporal window” for calculation of the signal STFT, and *τ* is a time-delay parameter that defines the STFT analysis time axis, and typically extends over the entire duration of the SUT. Finally, the SP of the SUT is given by the squared magnitude of the signal’s STFT, namely SP_*s*_(*τ*, *ω*_1_) = |STFT_*s*_(*τ*, *ω*_1_)|^2^.

As illustrated in Fig. 2, the proposed concept for TM-STFT involves processing the SUT with the cascade of two standard signal-processing units, namely

(i) A conventional temporal sampling unit where the SUT is modulated with a periodic train of short pulses^51^. In particular, the sampling pulse train *r*(*t*) follows the expression: $r\left( {t_1} \right) = \mathop {\sum }\nolimits_m p\left( {t_1 - mT_{\mathrm{R}}} \right)$, where *p*(*t*_1_) defines the temporal shape of each individual short pulse in the sampling sequence, and *T*_R_ holds for the repetition period, i.e., the sampling repetition frequency is *ω*_R_ = 2*π*/*T*_R_. We recall that for an ideal sampling process, one must ensure that the duration of the individual sampling pulses is shorter than the repetition period, i.e., Δ*t*_*p*_ < *T*_R_, whereas the repetition frequency must be larger than the full frequency bandwidth of the sampled signal *s*(*t*_1_), i.e., Δ*ω*_*s*_ < *ω*_R_. The latest condition is known as the Nyquist criterion^51^. The sampled signal at the output of this unit can be mathematically expressed as follows:
  6 $$s_{{\mathrm{sa}}}\left( {t_1} \right) 	= s\left( {t_1} \right) \times \mathop {\sum }\limits_m p\left( {t_1 - mT_{\mathrm{R}}} \right) \propto s\left( {t_1} \right) \times \mathop {\sum }\limits_q P\left( {q\omega _{\mathrm{R}}} \right)e^{\left( {iq\omega _{\mathrm{R}}t_1} \right)} \\ 	= {\, \mathop {\sum }\limits_q P\left( {q\omega _{\mathrm{R}}} \right)s\left( {t_1} \right)e^{\left( {iq\omega _{\mathrm{R}}t_1} \right)}} ,$$
  where ∝ holds for proportionality, and *m*,*q* = 0, ±1, ±2…, ±∞. In deriving this equation, we have simply used the Fourier series development of the periodic sampling pulse train^51^, where *P*(*ω*_1_) is the frequency spectrum of the individual sampling pulse, i.e., *P*(*ω*_1_) = FT{*P*(*t*_1_)}. The full-width spectral extent of *P*(*ω*_1_) is ∼Δ*ω*_*p*_. Notice that Eq. (6) ignores any potential latency in the sampling unit, an effect that is considered in our analysis at a later stage (see below). Each of the factors of the sum obtained in Eq. (6), i.e., $P\left( {q\omega _{\mathrm{R}}} \right)s\left( {t_1} \right)e^{\left( {iq\omega _{\mathrm{R}}t_1} \right)}$, corresponds to a copy of the input signal *s*(*t*_1_), frequency shifted by *qω*_R_ and weighted by the corresponding Fourier coefficient of the sampling pulse, *P*(*qω*_R_). Also recall that the Nyquist criterion ensures that each of these signal copies extend over a frequency bandwidth narrower than *ω*_R_, avoiding their spectral overlapping, see illustrated spectrum of the sampled signal in Fig. 2.
(ii) The sampling process in step (i) is followed by a frequency-dependent (or dispersive) time delay unit^51^, where the consecutive spectral copies of the sampled SUT, frequency spaced by *ω*_R_, are delayed by an amount Δ*τ* with respect to each other, as represented in Fig. 2. For simplicity, in the following analysis, we assume a positive delay as a function of the frequency shift (i.e., a positive slope of the delay vs. frequency curve or dispersion parameter) but similar results would be obtained for a negative dispersion parameter. Mathematically, the temporal waveform at the output of the dispersive delay unit can then be expressed as $g\left( {t_2} \right){\mathrm{e}}^{\left( { i\omega _0t_2} \right)}$, where *t*_2_ = *t*_1_ − Δ*t*_L_ defines the time variable at the system output, simply delayed with respect to the input by the latency or overall average delay of the system (including the latency in the sampling and dispersive delay units), Δ*t*_L_. The resulting temporal complex envelope of the output waveform is then
  7 $$g\left( {t_2} \right) 	\propto \mathop {\sum}\limits_q {P\left( {q\omega _{\mathrm{R}}} \right)s\left( {t_2 - q\Delta \tau } \right)e^{\left( {iq\omega _{\mathrm{R}}\left( {t_2 - q\Delta \tau } \right)} \right)}} \\ 	 { = \mathop {\sum}\limits_q {P\left( {q\omega _{\mathrm{R}}} \right)s\left( {t_2 - q\Delta \tau } \right)e^{\left( {iq\omega _{\mathrm{R}}t_2} \right)}e^{\left( { - iq^2\omega _{\mathrm{R}}\Delta \tau } \right)}} }.$$

Notice that in deriving Eq. (7) from Eq. (6) we have simply implemented the variable change: *t*_1_ ← *t*_2_ − *q*Δ*τ* to account for the delay that is introduced by unit (ii) on each of the spectral copies of the sampled input signal, i.e., a relative delay of *q*Δ*τ* for the *q*th spectral copy with respect to the original one (copy corresponding to *q* = 0, centered at *ω*_2_ = 0). Equation (7) shows that the waveform at the output of the two concatenated processing units, *g*(*t*_2_), consists of the coherent addition of a set of copies of the input SUT that are simultaneously shifted in time (with a relative delay between consecutive copies of Δ*τ*) and in frequency (with a relative frequency spacing between consecutive copies of *ω*_R_), while being weighted according to the frequency spectrum of the sampling pulses, namely, following the function *P*(*qω*_R_).

We further assume that the relative time delay in between consecutive spectral copies of the SUT is set to be equal to the sampling period, i.e., Δ*τ* = *T*_R_. Under this condition, the quadratic phase term in the latest sum in Eq. (7) is equal to unity, i.e., $e^{\left( { - iq^2\omega _{\mathrm{R}}\Delta \tau } \right)} \equiv 1$, for all values of *q*. In addition, the slope of the dispersive delay process (dispersion parameter) is then defined as

8 $$\left| {\ddot \phi } \right| = \frac{{\Delta \tau }}{{\omega _{\mathrm{R}}}} = \frac{{T_{\mathrm{R}}^2}}{{2\pi }}.$$

We reiterate that though the case of a positive dispersion parameter is considered in the detailed analysis presented here, similar results would be obtained for a negative dispersion parameter. To proceed further with our analysis, we will use the fact that the above-stated Nyquist criterion implies that the function *s*(*t*_2_) remains approximately constant over the temporal sampling period Δ*τ* = *T*_R_^51^. It is thus convenient to evaluate the resulting waveform from Eq. (7) over time slots of duration *T*_R_, namely, over each of the consecutive time slots defined by $nT_{\mathrm{R}} - \frac{{T_{\mathrm{R}}}}{2} \, \le \, t_2 \, < \, nT_{\mathrm{R}} + \frac{{T_{\mathrm{R}}}}{2}$, with *n* = 0, ± 1, ±2, … In the *n*th time slot, the function *s*(*t*_2_) can be approximated by *s*(*nT*_R_). The temporal waveform at the output of the dispersive delay unit along the *n*th time slot can then be expressed as follows:

9 $$g_n\left( {t^{\prime} } \right) 	\propto \mathop {\sum}\limits_q {P\left( {q\omega _{\mathrm{R}}} \right)s\left( {nT_{\mathrm{R}} - qT_{\mathrm{R}}} \right)e^{\left( {iq\omega _{\mathrm{R}}t^{\prime} } \right)}} \\ 	= { e^{\left( {in\omega _{\mathrm{R}}t^{\prime} } \right)}\mathop {\sum}\limits_{q^{\prime} } {P\left( {\left[ {n - q^{\prime} } \right]\frac{{T_{\mathrm{R}}}}{{\ddot \phi }}} \right)s\left( {q^{\prime} T_{\mathrm{R}}} \right)} \,e^{\left( { - i\omega _1q^{\prime} T_{\mathrm{R}}} \right)}} ,$$

where we have implemented the following variable changes: *t*′ = *t*_2_ − *nT*_R_, *q*′ = *n* − *q* (thus, *q*′ = 0, ±1, ±2, …, ±∞), and $\omega _1 = t^{\prime} /\ddot \phi$. We define now the following new function:

10 $$h\left( t \right) = P\left( {\omega _1 = - t/\ddot \phi } \right),$$

which will be referred to as the “temporal window function”. Introducing this newly defined function in Eq. (9), we obtain:

11 $$g_n\left( {t^{\prime} } \right) \propto e^{\left( {in\omega _{\mathrm{R}}t^{\prime} } \right)}\mathop {\sum}\limits_{q\prime } {h\left( {q^{\prime} T_{\mathrm{R}} - nT_{\mathrm{R}}} \right)s\left( {q^{\prime} T_{\mathrm{R}}} \right)\,e^{\left( { - i\omega _1q^{\prime} T_{\mathrm{R}}} \right)}}.$$

The sum in Eq. (11) can be interpreted as the discretized version of the corresponding continuous-time integral form, sampled at *t*_1_ ← *q*′*T*_R_, and in particular, within the defined analysis temporal slot (−*T*_R_/2 ≤ *t*′ < *T*_R_/2), the expression in Eq. (11) is then equivalent to:

12 $$g_n\left( {t^{\prime} } \right) \propto e^{\left( {in\omega _{\mathrm{R}}t^{\prime} } \right)} \int _{ - \infty }^\infty h\left( {t_1 - nT_{\mathrm{R}}} \right)s\left( {t_1} \right)e^{\left( { - i\omega _1t_1} \right)}\,dt_1,$$

where we recall that $\omega _1 = t^{\prime} /\ddot \phi$. The equivalence between the discretized and continuous-time versions of the same expression in Eqs. (11) and (12) is based on the fact that the temporal sampling process used to establish this equivalence, *t*_1_ ← *q*′*T*_R_, satisfies the Nyquist criterion in regard to both the input SUT, *s*(*t*_1_) (as per the specifications of the sampling unit (i)), and the newly defined temporal window function *h*(*t*_1_). Notice that following the definition in Eq. (10), sampling the window function *h*(*t*_1_) at *q*′*T*_R_ corresponds to sampling the pulse frequency spectrum, *P*(*ω*_1_), at *q*′*ω*_R_, and this process strictly satisfies the related Nyquist criterion, considering that the pulse temporal width is necessarily shorter than the sampling period, Δ*t*_*p*_ < *T*_R_. Finally, Eq. (12) involves a Fourier integral, as defined in Eq. (4) above, so it can be re-written as follows:

13 $$\begin{array}{*{20}{c}} {g_n\left( {t_2} \right) \propto e^{\left( {in\omega _{\mathrm{R}}t_2} \right)} \times {\mathrm{FT}}\left\{ {h\left( {t_1 - nT_{\mathrm{R}}} \right)s\left( {t_1} \right)} \right\}_{\omega _1 = \frac{{t_2 - nT_{\mathrm{R}}}}{{\ddot \phi }}}} \\ { = e^{\left( {in\omega _{\mathrm{R}}t_2} \right)} \times {\mathrm{STFT}}_s\left( {nT_{\mathrm{R}},\omega _1 = \frac{{t_2 - nT_{\mathrm{R}}}}{{\ddot \phi }}} \right),} \end{array}$$

where we have used the definition of STFT in Eq. (5). As detailed in the main text, Eq. (13) shows that except for a linear phase term, $e^{\left( {in\omega _{\mathrm{R}}t_2} \right)}$, the output temporal waveform, when evaluated at each time slot $nT_{\mathrm{R}} - \frac{{T_{\mathrm{R}}}}{2} \, \le \, t_2 \, < \, nT_{\mathrm{R}} + \frac{{T_{\mathrm{R}}}}{2}$, is proportional to a TM copy of the FT of the SUT truncated by a temporal window centered at *nT*_R_. The frequency-to-time scaling factor is given by the dispersive delay slope, $\ddot \phi$, as defined in Eq. (8). The temporal window of the conducted SP analysis is determined by the function in Eq. (10), namely a TM copy of the sampling pulse spectrum. Said other way, the output temporal waveform follows the STFT of the SUT, evaluated at the sampling points implemented by unit (i). Thus, the intensity of the output temporal waveform is proportional to a full SP or time–frequency energy distribution of the SUT, i.e., $\left| {g_n\left( {t_2} \right)} \right|^2 \propto {\mathrm{SP}}_s( {nT_{\mathrm{R}},\omega _1 = \frac{{t_2 - nT_{\mathrm{R}}}}{{\ddot \phi }}} )$.

### Limitation of the TM-FT method for continuous waveforms

The TM-FT of a time-limited signal with a duration Δ*t*_*s*_ requires stretching the signal with a group-velocity dispersion larger than $\left| \ddot \phi _{{\mathrm{TM}} - {\mathrm{FT}}} \right| \, > \, \Delta t_s^2$. As a result, the input signal is temporally stretched to an output duration $\Delta t_{{\mathrm{out}}} \approx \left| \ddot \phi _{{\mathrm{TM}} - {\mathrm{FT}}}\right|\times \Delta \omega _s \, > \, \Delta t_s \times {\mathrm{TBP}}$, where Δ*ω*_*s*_ is the full frequency bandwidth of the input signal and TBP ≈ Δ*t*_*s*_ × Δ*ω*_*s*_ defines the number of resolvable points in the spectrum analysis. Thus, in the case of a continuous incoming signal, for each analyzed signal portion with a duration Δ*t*_*s*_, one misses the signal information over the rest of an entire section longer than Δ*t*_out_ > Δ*t*_*s*_ × TBP. As a result, the percentage of lost information can be estimated to be at least [(TBP − 1)/TBP] × 100%. For instance, for a TM-FT analysis with a relatively modest number of resolvable points (TBP ~10), about 90% of the signal information is necessarily lost in the analysis.

### Photonic implementation of the TM-SP analog-processing system for RT-SA of microwave signals

The user-defined microwave SUTs used for testing the photonics-based TM-SP setup (Figs. 3–5) are all generated from an electronic AWG (Tektronix AWG7122C) with an analog bandwidth of 10 GHz and a sampling rate of 24 GS/s. The optical sampling pulses used for sampling the microwave SUTs (results in Figs. 3–5) are generated from an actively mode-locked fiber laser (Pritel—Ultrafast Optical Clock), with each individual pulse having a Gaussian-like temporal shape with a FWHM time width of Δ*t*_*p*_ ≈ 7 ps (corresponding transform-limited amplitude FWHM frequency bandwidth Δ*ω*_*p*_ ≈ 2*π* × 140 GHz), and a repetition period *T*_R_ ≈ 205.76 ps (sampling rate = 4.86 GHz). Temporal sampling of the microwave SUTs is performed with a 40-GHz electro-optic MZM biased at *V*_*π*_ = 5.5V. An optical polarization controller is used before the MZM to optimize the electro-optic modulation process. The MZM performs carrier-suppressed amplitude modulation of the microwave SUT within its linear region of operation, for SUTs with peak-to-peak voltage amplitude (*V*_p-p_) up to 4 V. The sampling rate of 4.86 GHz enables the analysis of microwave SUTs with a full frequency bandwidth up to ∼4.86 GHz (Nyquist criterion). Subsequently, in the two evaluated platforms, the modulated optical pulses are linearly propagated through a DCF, which provides a second-order dispersion amount of ∼6825 ps^2^ rad^−1^, about 1.3% off the ideal amount of dispersion from Eq. (2), $\ddot \phi = T_{\mathrm{R}}^2/2\pi = \;6738.2$ ps^2^ rad^−1^, over the full frequency bandwidth of the sampling pulses. The total loss of the DCF used in the experiments is 28 dB, which is compensated for using an optical pulse amplifier (Pritel EDFA) before photo-detection. The output non-repetitive waveform is captured with a 50 GHz bandwidth photo-detector attached to a 63-GHz bandwidth real-time oscilloscope (Keysight DSAZ634A), with no averaging. The real-time oscilloscope is triggered with a marker from the electronic AWG that coincides with the beginning of the microwave modulation signal. No synchronization is needed between the optical pulses and microwave signal in the modulation process. Further processing of the captured temporal traces, namely resampling, retiming, and rescaling to the frequency domain, is performed numerically offline, using Matlab in a personal computer. Note that the measured output waveform is sampled in the real-time scope at a rate of 160 GS/s or with a sampling period of 6.25 ps. This translates into a non-integer number of samples, i.e., 32.922 samples, per analysis period (*T*_R_ ≈ 205.76 ps) along the corresponding TM frequency axis, leading to the vertical frame displacements that can be observed, period to period, in the 2D SP representations in Figs. 3–5. These frame displacements along the vertical frequency axis are also partly attributed to the finite timing jitter in the picosecond optical pulses used for temporal sampling. Re-sampling was still performed to ensure an integer number of samples (100) per analysis period to facilitate the 2D representations.

### SP simulations

The simulated SP distributions shown in Fig. 4 are computed using a numerical algorithm in Matlab. The results in Fig. 4 show the numerical SP (squared magnitude of the STFT) of the corresponding digitized measured input SUT. The sampling period of the discretization used in simulations is fixed to be the same as the sampling period of the optical pulses (*T*_R_ ≈ 205.76 ps) used in the experiments. Hence, the number of samples along the time and frequency axis are identical for all the 2D distributions (numerical and experimental) shown in Fig. 4. The analysis temporal window, *h*(*t*), in the proposed TM-SP method is given by the TM version of the sampling pulse spectrum. The sampling optical pulses in the demonstrated experimental setup have a Gaussian-like shape; therefore, the corresponding pulse spectrum is also Gaussian in shape, with an estimated TM amplitude FWHM of ~5.9 ns. Consistently, for the numerical SP calculations, a Gaussian temporal analysis window with a FWHM time-width of 5.9 ns is also used. The speed of calculation of each simulated SP is quantified as the number of FFTs that are computed per second, which is 6 FFT/s with our available computation power. All the simulations were performed in MATLAB 2015a running on a Windows 10, 64-bit operating system.

## Supplementary information

Supplementary Information

## Data Availability

The data that support the plots within this paper and other findings of this study are available from the corresponding author upon reasonable request.
