# Supplementary material for: Real-time gap-free dynamic waveform spectral analysis with nanosecond resolutions through analog signal processing
Source: Nat Commun. 2020 Jul 3;11:3309. doi: 10.1038/s41467-020-17119-2 (PMC7335167; doi:10.1038/s41467-020-17119-2)
Supplement: Supplementary file 1 — Supplementary Information [file 41467_2020_17119_MOESM1_ESM.pdf]

Real-time gap-free dynamic waveform spectral analysis with nanosecond resolutions through analog signal processing

Saikrishna Reddy Konatham[1], Reza Maram[1], Luis Romero Cortés[1], Jun Ho Chang[2], Leslie Rusch[2], Sophie LaRochelle[2], Hugues Guillet de Chatellus[1,3], and José Azaña[1,*]

1. Institut National de la Recherche Scientifique – Énergie, Matériaux et Télécommunications (INRS-EMT), 800 de la Gauchetière Ouest, Suite 6900, H5A 1K6, Montréal, Québec, Canada
2. Centre for Optics, Photonics and Lasers (COPL), Department of Electrical and Computer Engineering, Université Laval, Quebec, G1V 0A6, Canada
3. Université Grenoble Alpes/CNRS, LIPHY, F-38000 Grenoble, France

*azana@emt.inrs.ca

Supplementary information:

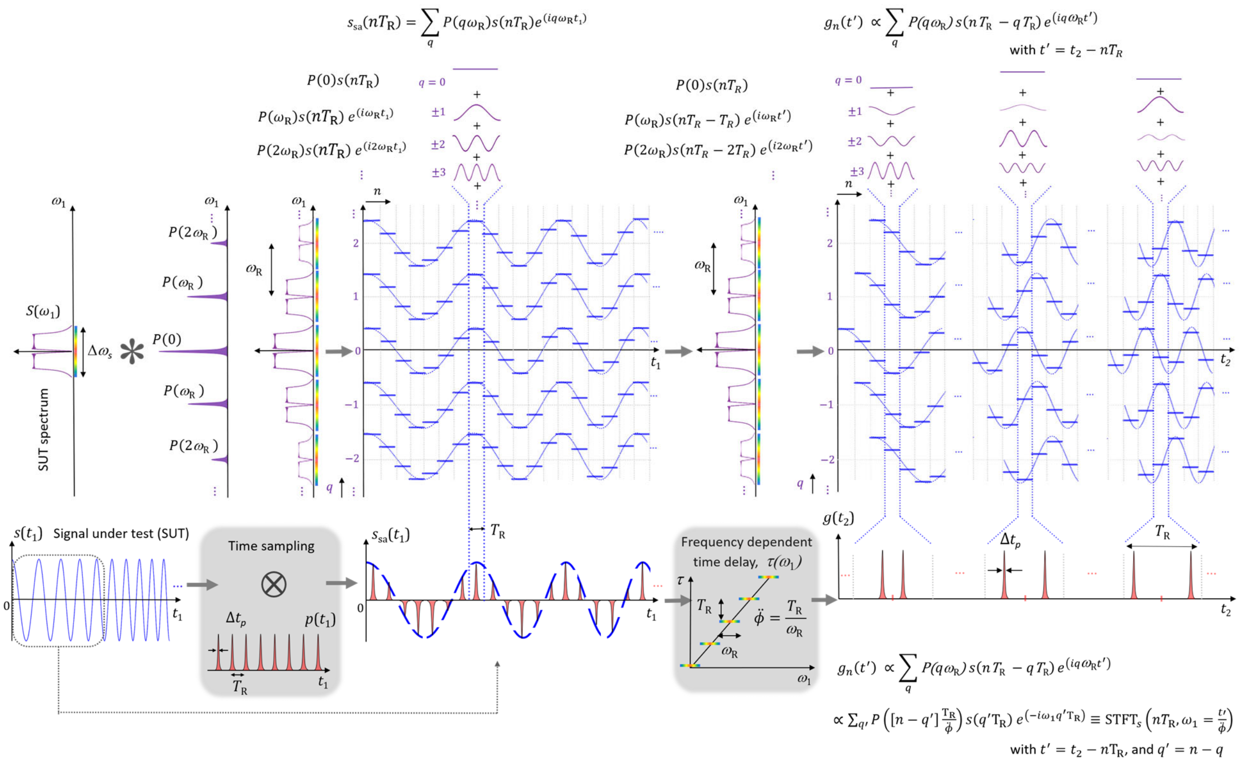

Supplementary Fig. 1. Illustration of the mathematical relationships that lead to the analog computation and time mapping of the STFT of the incoming SUT performed by a combination of temporal sampling and dispersive delay. The temporal sampling of the SUT with a periodic train of short pulses create non-overlapping frequency shifted copies (purple color) of the SUT spaced by ω_R along the vertical frequency axis. The temporally sampled SUT is represented by discrete samples (blue color) along the horizontal time axis, with sample spacing $T_R = 2\pi/\omega_R$. The time-domain profile of the sampled SUT along each sampling period of duration T_R can be interpreted as the coherent summation of a set of harmonically related frequencies (0, ω_R, $2\omega_R$ …) that are weighted by the sampling pulse frequency spectrum and the (constant) SUT complex amplitude value at the considered sampling point. The subsequent frequency-dependent (or dispersive) time delay process delays the adjacent frequency shifted copies of the SUT, with spectral spacing ω_R, by T_R with respect to each other, as represented in the output time-frequency plane. This induces a coherent addition of the harmonically related frequency components (0, ω_R, $2\omega_R$ …) along each sampling-period slot (duration T_R) in such a way that these different frequency components are now weighted by the sampling pulse spectrum *and* the changing complex amplitudes of a set of consecutive time samples of the SUT, leading to the calculation of the FT of the corresponding time-windowed section of the SUT. Thus, the sampling pulse spectrum determines the extension of the analyzed signal section along each sampling-period slot, and the analyzed section is in fact shifted by T_R from each sampling-period slot to the following one. This ultimately leads to the calculation of a running time-windowed FT, or STFT, of the input SUT. See Methods section for the detailed mathematical derivations.

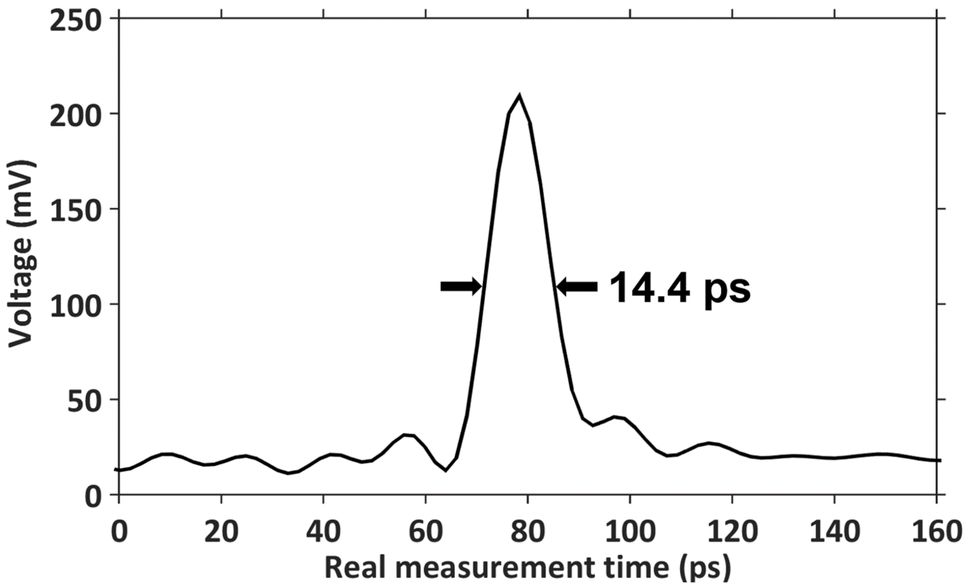

Supplementary Fig. 2. A sample of the sampling optical pulses that are directly measured in a 63-GHz real-time scope following detection with a 50-GHz photodetector. The intensity FWHM of the detected pulses is Δt_{d} ~ 14.4 ps. This is the value used to estimate the frequency resolution of the implemented real-time SP analysis.

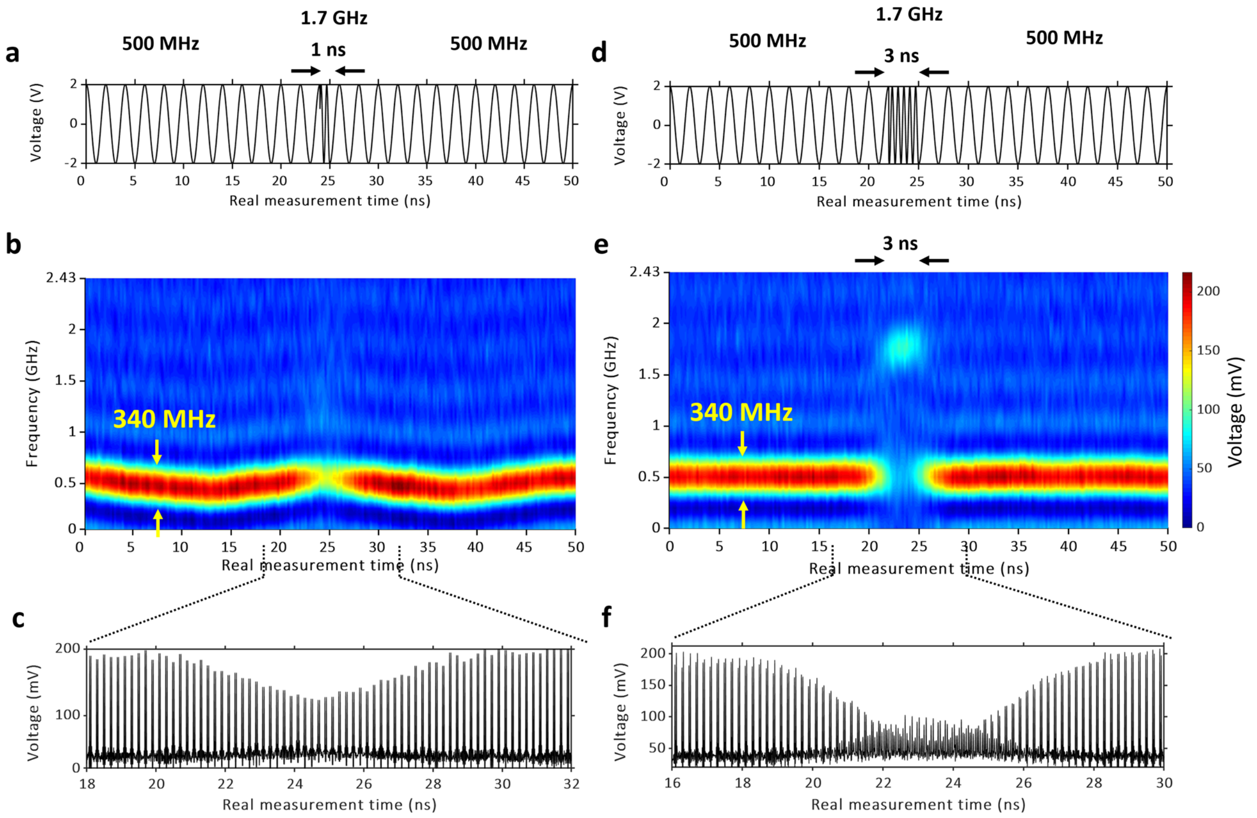

Supplementary Fig. 3. Experimental results for evaluation of joint time-frequency resolutions of the time-mapped spectrogram analysis. The photonic sampling and delay system with the specifications described in the text is used for spectrogram analysis of the microwave signals shown in the top plots. Each of the analyzed signals, **Supplementary Figs. 3(a) and (d),** consists of a high-frequency (1.7-GHz) sinusoid in between two low-frequency (500 MHz) sinusoid, the difference being in that the 1.7-GHz sinusoid in the left signal, **Supplementary Fig. 3(a),** exhibits a shorter duration (1 ns) than that of the signal to the right (3 ns), **Supplementary Fig. 3(d)**. The bottom plots, **Supplementary Figs. 3(b) and (e),** respectively show the 2D spectrogram distributions of the two analyzed signals that are recovered from the measured waveforms at the output of the sampling & delay system. A time resolution of ~5.9 ns (FWHM width) is estimated for the photonic sampling and dispersion configuration under test. Note the frequency resolution is modified to ~ $2\pi\times340$ MHz due to limited photo-detection bandwidth. As expected, this configuration is unable to resolve the 1-ns-long high-frequency event of the first signal, **Supplementary Fig. 3(b)**, but it can resolve the longer (3-ns) high-frequency event in the second signal with a sufficient amplitude, **Supplementary Fig. 3(e),** consistently with the theoretical time resolution limitations. **Supplementary Fig. 3(c) and (f)** show the transition of the time-mapped spectra as the microwave signal under test changes from low frequency (500 MHz) to high-frequency (1.7 GHz) in each of the test case, respectively.

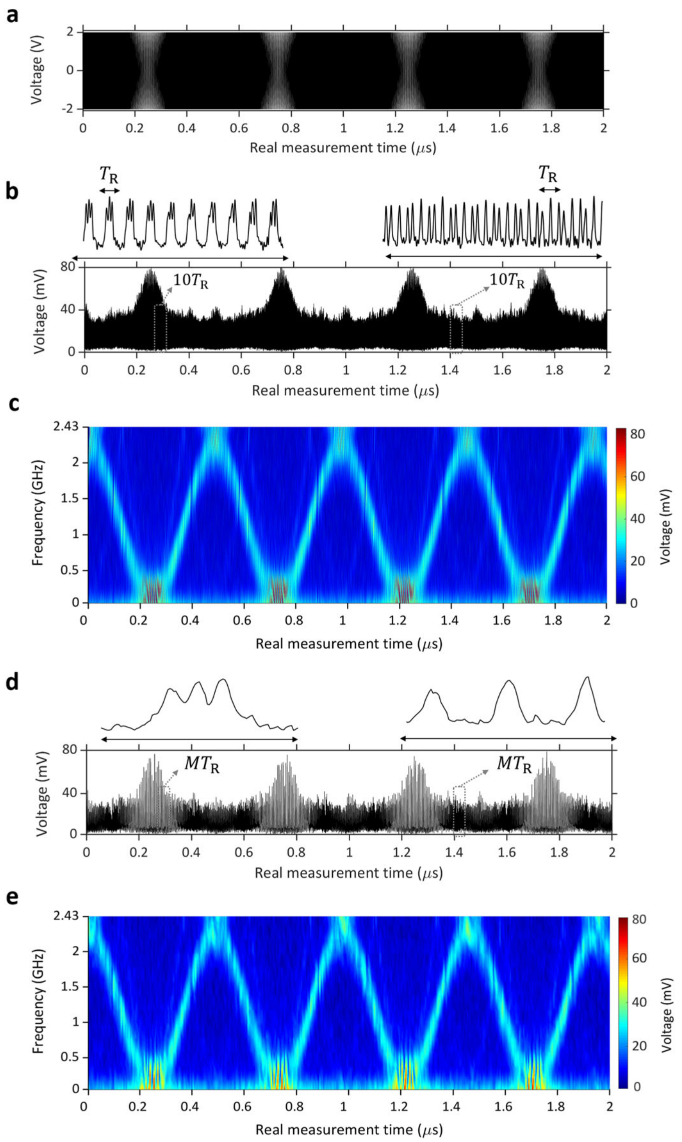

Supplementary Fig. 4. Experimental results showing the oversampling features in the time-mapped spectrogram analysis method. Supplementary Fig. 4(a) shows the input SUT: a 2 MHz tone that is frequency modulated on a 1.215-GHz carrier with a maximum deviation of ±1.215 GHz. These are results obtained with the same photonic sampling and dispersive system that was used for TM-SP analysis of high-speed microwave signals, scheme shown in Fig. 3(a). **Supplementary Fig. 4(b)** shows the temporal waveform at the output of the TM-SP system, showing the mapping of the input frequency spectrum along each time slot of duration T_{R} (sampling period), repeating over M consecutive slots, where M is the sampling period to pulse width ratio, given by $M = T_{\mathrm{R}}/\Delta t_p \sim 29$ in the present experimental design (only 10 consecutive analysis periods shown in the plot). **Supplementary Fig. 4(c)** shows the 2D spectrogram distribution that is directly mapped from the measured temporal trace at the system output. Note that the frequency axis can be resolved by M distinct frequency bins, but in the current experimental configuration, the increase in frequency resolution due to limited detection-bandwidth leads to only $T_{\mathrm{R}}/\Delta t_{\mathrm{d}} \sim 14$ distinct frequency bins. Only the positive frequency side is shown here to facilitate interpretation. **Supplementary Fig. 4(d):** The effective oversampling of the spectrogram information shown in (b) is advantageous in designing the detection stage of the obtained time-frequency distribution. Using this property, the obtained spectrogram could be fully retrieved by sampling the output waveform at a significantly relaxed rate, just slightly below the original sampling rate of 4.69 GS/s, i.e., with a period equal to $T_{\mathrm{R}} + (T_{\mathrm{R}}/M) \sim 212.9$ ps. **Supplementary Fig. 4(e)** shows the 2D spectrogram distribution mapped from the down-sampled output temporal trace, in excellent agreement with the 2D spectrogram distribution that is directly retrieved from the over-sampled output trace.
